# An Empirical Study of Open-Set Gesture Recognition on WiFi BVP Features

**DOI:** 10.3390/s26216723

**Published:** 2026-10-23

**Authors:** Zhenzhen Zhao, Heng Wang

**Affiliations:** 1 School of Computer and Information Engineering (School of Software), Henan University of Economics and Law, Zhengzhou 450046, China; 2 College of Mechanical and Electrical Engineering, Henan Agricultural University, Zhengzhou 450002, China; dawn.wangh@henau.edu.cn

**Keywords:** open-set recognition, WiFi sensing, gesture recognition, Body-coordinate Velocity Profile (BVP), domain-disjoint evaluation, classifier decision boundary

## Abstract

**Highlights:**

**What are the main findings?**

- On the CNN–GRU backbone, closed-set Softmax classifiers consistently misclassify 49.7–74.3% of unknown gestures with high confidence (>0.8), even though the same backbone achieves 76.9–84.3% accuracy on known classes;
- The closed-set high-confidence misclassification rate is strongly associated with open-set AUROC ($R^{2}$ = 0.918, *p* = 0.0026), whereas the embedding-space geometric distance between unknown and known classes shows no significant association ($R^{2}$ = 0.018, *p* = 0.80);
- No single OSR method consistently outperforms all others across unknown gestures, and frozen and fine-tuned backbones yield comparable open-set performance (typically within ±0.02 AUROC).

**What are the implications of the main findings?**

- Open-set difficulty on BVP features appears to be more closely related to classifier-level decision-boundary confusion than to geometric separation, suggesting that future OSR methods should target classifier uncertainty rather than feature-space geometry;
- The results provide a systematic benchmark of seven OSR methods on WiFi BVP features and show that fine-tuning a pre-trained backbone does not consistently improve open-set performance;
- Multi-objective evaluation across different operating points offers practical guidance for method selection under varying deployment constraints.

**Abstract:**

WiFi-based gesture recognition using domain-independent Body-coordinate Velocity Profile (BVP) features has demonstrated strong cross-domain generalization, but existing methods generally rely on closed-set assumptions, limiting their use in open-world scenarios. In this paper, we present a systematic empirical study of open-set gesture recognition on WiFi BVP features. On the CNN–GRU backbone, we evaluate seven representative OSR methods—MSP, OpenMax, Energy, ODIN, K-NN, prototype, and rejection head—under two backbone strategies (frozen and full fine-tuning), across six unknown gestures and three random seeds. Our experiments show that closed-set Softmax classifiers consistently misclassify unknown gestures with high confidence (49.7–74.3% across six gestures), and that the closed-set high-confidence misclassification rate is strongly associated with open-set AUROC ($R^{2}$ = 0.918, *p* = 0.0026), whereas embedding-space geometric distance is not ($R^{2}$ = 0.018, *p* = 0.80). This suggests that open-set difficulty may stem from classifier decision-boundary confusion rather than geometric separation. We further find that no single OSR method consistently outperforms all others across unknown gestures, and that frozen and fine-tuned backbones yield comparable open-set performance (typically within ±0.02 AUROC). Finally, we provide multi-objective evaluation and deployment observations, including Pareto frontiers and multiple operating points. These results offer a systematic benchmark for open-set WiFi gesture recognition and highlight the importance of classifier-level factors in determining open-set difficulty.

## 1. Introduction

WiFi-based gesture recognition is a technology that uses ubiquitous wireless signals to sense, capture, and analyze human hand gestures. When a person moves within the coverage area of a WiFi signal, limb movements block, reflect, or refract radio waves, altering the Channel State Information (CSI) of the signal, which can be used to infer gestures [1]. This approach requires no additional sensing devices and enables contactless, secure, and privacy-preserving gesture recognition [2,3], making it attractive for applications such as vital sign monitoring [4,5] and activity recognition [6,7,8].

Early WiFi-based sensing systems for human activity and gesture recognition relied on handcrafted features extracted from CSI, such as amplitude waveforms and Doppler shift profiles, combined with conventional machine learning classifiers [6,7]. However, these features inevitably encode static environmental characteristics, causing recognition performance to degrade when deployment environments, user positions, or body orientations change. To improve robustness, deep learning-based approaches have been proposed to automatically learn discriminative representations from CSI data. WiGRUNT employs a dual-attention network to capture spatial and temporal gesture characteristics [9], while MKFi achieves temporally robust activity recognition under limited labeled data [8]. Domain generalization methods have further been developed to alleviate domain-shift issues, with Widar3.0 introducing the Body-coordinate Velocity Profile (BVP), a domain-independent representation that is largely invariant to room geometry and user position [10]. Cross-domain invariant feature learning [11,12] and prototype-based learning [13,14] have also been explored to improve generalization. However, all of these methods operate under the closed-set assumption: every gesture class encountered during deployment is assumed to have been seen during training. In practical scenarios, users may perform novel gestures that were not present in the training set, and closed-set models often force such novel gestures into known categories, producing unreliable predictions.

Addressing open-set recognition (OSR) in the WiFi gesture domain remains largely unexplored. WiOpen first introduced open-set WiFi gesture recognition by modeling sample-embedding relationships through an uncertainty quantification network to dynamically construct open-set decision boundaries [15]. More recently, Wi-CO combines episodic meta-training with an open-set rejection module that estimates the novelty of query samples relative to the support set, enabling simultaneous adaptation to domain shifts and unknown gesture classes [16]. However, both methods operate directly on raw CSI signals, which are highly susceptible to environmental contamination and tend to entangle gesture-related characteristics with user identity, location, and body orientation. Such coupling may limit generalization across unseen deployment conditions and reduce the reliability of unknown gesture detection.

**Figure 1:**
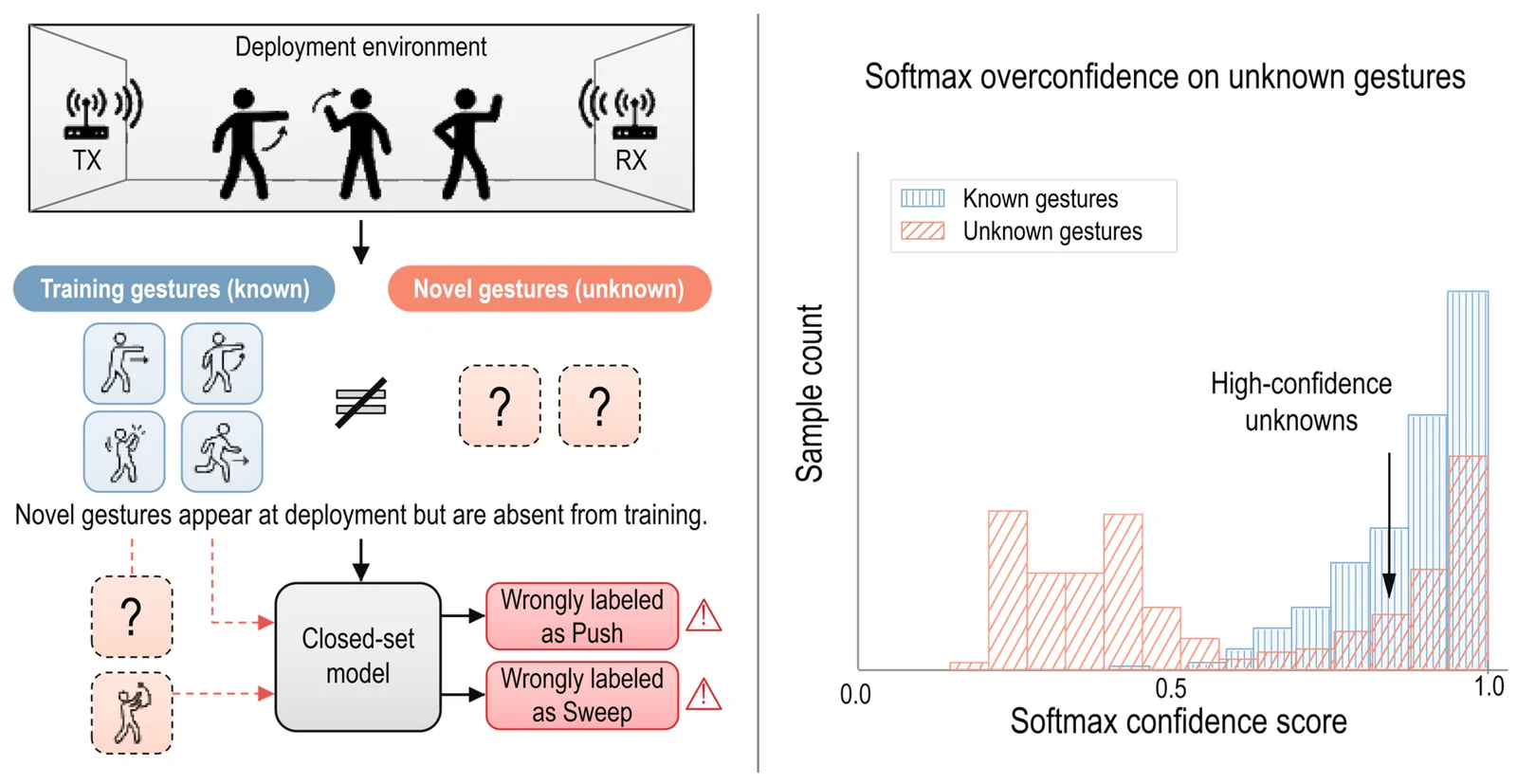
Motivation and overview. (**Left**): Known and unknown gestures are fed into a closed-set classifier, which confidently misclassifies unknown gestures into known categories. (**Right**): Distribution of maximum Softmax probabilities for known-class (blue) and unknown-class (red) test samples, showing that a substantial fraction of unknown gestures receive high confidence scores (>0.8), overlapping with the known-class distribution.

To address these challenges, we present an empirical study of open-set gesture recognition on the domain-independent WiFi BVP feature space. Figure 1 illustrates the problem: when novel gestures appear at deployment, a closed-set classifier tends to misclassify them into known categories with high confidence. To systematically examine this problem, we evaluate seven representative OSR methods—MSP [17], OpenMax [18], Energy [19], ODIN [20], K-NN [10], prototype [21], and rejection head [22,23]—on top of a shared CNN–GRU backbone. We consider two backbone strategies: frozen and full fine-tuning. Under a strict domain-disjoint evaluation protocol on the Widar3.0 BVP dataset [10], we evaluate these methods across six unknown gestures and three random seeds. Our results reveal that closed-set Softmax classifiers consistently misclassify unknown gestures with high confidence, and that open-set performance is associated with classifier-level confusion rather than geometric separation. We further show that no single method consistently outperforms all others across unknown gestures, and that fine-tuning the backbone does not consistently improve open-set performance.

The main contributions of this paper are as follows:

1. We present an empirical study of open-set gesture recognition on WiFi BVP features, evaluating seven representative OSR methods under two backbone strategies (frozen and full fine-tuning) across six unknown gestures and three random seeds on the CNN–GRU backbone.
2. We find that closed-set Softmax classifiers consistently misclassify unknown gestures with high confidence (49.7–74.3%), and that the closed-set high-confidence misclassification rate is strongly associated with open-set AUROC ($R^{2}$ = 0.918, *p* = 0.0026), whereas embedding-space geometric distance is not ($R^{2}$ = 0.018, *p* = 0.80). This suggests that open-set difficulty may stem from classifier decision-boundary confusion rather than geometric separation.
3. We show that no single OSR method consistently outperforms all others across unknown gestures, and that frozen and fine-tuned backbones yield comparable open-set performance (typically within ±0.02 AUROC), challenging the assumptions that fine-tuning always helps and that a universal optimal OSR method exists.
4. We provide multi-objective evaluation and deployment observations, including Pareto frontiers, multiple operating points (95%/90%/80%/70% acceptance rates), and a reference table for method selection under different deployment constraints.

The remainder of this paper is organized as follows. Section 2 reviews related work on closed-set WiFi gesture recognition, existing open-set recognition methods, and prototype networks for WiFi sensing. Section 3 presents the evaluation framework and the seven OSR methods. Section 4 describes the experimental setup, reports the main results, analyzes the factors associated with open-set difficulty, and provides multi-objective evaluation and discussion. Section 5 concludes the paper and summarizes the main findings.

## 2. Related Work

### 2.1. Closed-Set WiFi Gesture Recognition

WiFi-based human activity and gesture recognition methods have evolved from closed-set models to cross-domain and few-shot learning approaches. Early closed-set methods typically adopted conventional classifiers on handcrafted CSI features [6,7]. To address their sensitivity to environmental changes, deep learning approaches such as WiGRUNT [9] and MKFi [8] were introduced to learn more robust representations. Nevertheless, these deep learning approaches still assume that training and testing data follow similar distributions, leaving them vulnerable to domain shifts.

Different strategies have been proposed to improve cross-domain robustness. Widar3.0 introduces the Body-coordinate Velocity Profile (BVP), a domain-independent representation that is largely invariant to room geometry and user position [10]. Domain generalization methods such as AirFi [11] and domain redefinition approaches [12] learn environment-invariant or latent-domain-invariant representations from multiple source environments. Similar cross-domain strategies have also been explored for WiFi-based gait recognition [24,25]. Although these methods improve cross-domain robustness, they still rely on predefined gesture categories and labeled training data.

To enhance generalization under limited supervision, few-shot and meta-learning techniques have been introduced for WiFi gesture recognition. OneFi addresses one-shot recognition of unseen gestures using commodity WiFi devices [26]. Prototype networks [21] represent each class by a prototype vector in a learned embedding space, enabling classification via nearest-prototype matching. Prototype-based methods have also been applied to WiFi gesture recognition [13,14,27]; we discuss them in detail in Section 2.3.

Despite these advances, all existing approaches operate under the closed-set assumption, where every gesture category encountered during deployment is known during training. In practical deployments, users may perform entirely novel gestures absent from the training set. Existing closed-set models lack explicit mechanisms for detecting and rejecting such unknown gestures, often forcing them into known categories and resulting in unreliable predictions. The idea of abstracting task-relevant behavioral characteristics from content-dependent variations has been explored in other domains. For example, Ref. [28] investigates how handwriting style can be abstracted from written content, which is conceptually related to the use of BVP representations for suppressing environmental and domain-specific factors in WiFi sensing.

### 2.2. Open-Set Recognition

Open-set recognition (OSR) addresses the practical scenario in which a model must not only recognize known classes but also identify samples belonging to previously unseen classes during deployment. The simplest and most widely adopted baseline is the maximum Softmax probability (MSP) method [17], which rejects samples whose maximum Softmax probability falls below a predefined threshold. However, MSP often fails to provide reliable rejection signals because the Softmax normalization property forces the model to distribute probability mass among known classes even for inputs that do not belong to any of them. OpenMax [18] extends the Softmax classifier by fitting per-class Weibull distributions to penultimate-layer features and reallocating probability mass to an unknown class. Subsequent methods have explored alternative scoring functions and feature representations. ODIN [20] applies temperature scaling and input preprocessing to amplify the separation between known- and unknown-class scores without retraining. Energy-based OOD detection [19] uses the energy score derived from logits as a more effective alternative to Softmax probability. Prototype networks [21] represent each known class by a prototype vector in the embedding space and use the distance to the nearest prototype as the rejection score. Pseudo-unknown-based methods, such as OpenGAN [22] and Outlier Exposure [23], train an auxiliary rejection module using generated or external outliers. Mixup [29] is a common technique for generating pseudo-unknown samples by interpolating between different known classes. ARPL [30] introduces adversarial reciprocal-point learning, where each known class is associated with learnable reciprocal points that define its boundary in the feature space. These methods have substantially advanced open-set recognition in computer vision, but their effectiveness on WiFi sensing signals remains underexplored.

In the WiFi sensing domain, open-set recognition remains in a nascent stage. Widar3.0 [10] introduced a K-NN-based outlier detection algorithm that identifies unknown gestures by comparing the local density of a test sample to the expected density of resident samples. WiOpen [15] first denoises CSI measurements using the CSI ratio and then models the relationships among sample embeddings through an uncertainty quantification network, thereby dynamically constructing open-set decision boundaries for individual samples. More recently, Wi-CO [16] learns domain-agnostic feature representations through episodic meta-training and incorporates an open-set rejection module that estimates the novelty of query samples relative to the support set. Despite these advances, both WiOpen and Wi-CO are designed for CSI signal spaces rather than domain-independent BVP features. Since CSI measurements are highly susceptible to environmental contamination, the learned representations may contain substantial noise and environment-specific information, which can adversely affect recognition performance. Furthermore, gesture-related characteristics are often entangled with user identity, location, and body orientation in raw CSI representations. Such coupling may limit the model’s ability to generalize across unseen deployment conditions and reliably distinguish unknown gesture classes. Physical-layer features in WiFi systems may exhibit unexpected sensitivity when the deployment distribution or signal characteristics change [31]. This further motivates the need for open-set recognition mechanisms that can explicitly reject inputs from unseen distributions.

### 2.3. Prototype Networks for WiFi Sensing

Prototype networks were originally proposed by Snell et al. [21] for few-shot learning, where a classifier must generalize to new classes given only a small number of examples per class. The key idea is to learn a metric space where each class is represented by a prototype vector, typically the mean of the embedded support examples, and classification is performed by assigning a query sample to the nearest prototype in this space. The distance-based classification mechanism of prototype networks also provides a natural rejection score, which has been exploited in computer vision for open-set recognition [32].

In WiFi sensing, prototype networks have been primarily adopted for cross-domain and few-shot gesture recognition. ModProto [27] extends the standard prototype network with class-conditional domain-alignment objectives, learning prototype representations that are simultaneously discriminative across gesture classes and invariant across environmental domains. The dual-path prototypical network [13] separates gesture-discriminative and domain-invariant features into dual-processing branches and performs prototype-level feature aggregation to improve cross-domain generalization. ProFi-Net [14] employs curriculum-based augmentation and progressive training on increasingly difficult samples to enhance the quality of prototype representations for few-shot WiFi gesture recognition. These methods collectively demonstrate that prototype-based learning is an effective paradigm for learning compact, domain-agnostic feature representations from WiFi signals, but they all operate under the closed-set assumption.

However, existing prototype-based methods in WiFi sensing operate exclusively under the closed-set assumption, where the set of gesture classes remains fixed across training and testing. They focus on improving closed-set classification accuracy across domains, rather than addressing the open-set scenario where novel gesture classes may appear during deployment. The distance-based rejection capability inherent to prototype networks has been widely exploited in computer vision for open-set recognition, but its effectiveness on WiFi BVP features remains underexplored.

In this study, prototype is evaluated as one of seven OSR methods on BVP features. Unlike prior prototype-based methods that learn both the backbone and prototypes jointly for closed-set classification, we keep the backbone frozen after closed-set pre-training and learn only the prototype vectors under the frozen strategy; in the full FT setting, the backbone is fine-tuned together with the prototypes. This design is motivated by our empirical observation that backbone fine-tuning does not consistently improve open-set performance on BVP features. The Euclidean distance to the nearest prototype serves as the rejection score, without requiring an auxiliary rejection head or self-supervised perturbation training.

## 3. Framework and Methods

### 3.1. Framework Overview

**Figure 2:**
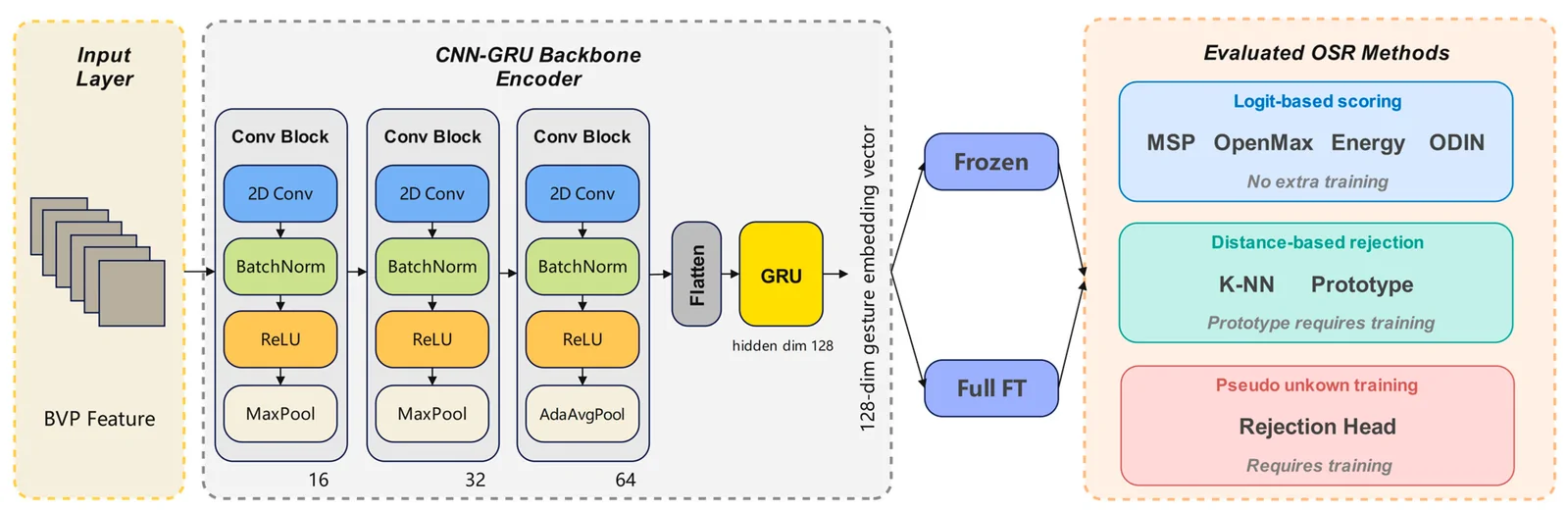
Overview of the evaluation framework. A CNN–GRU backbone is pre-trained on the closed-set classification task. Seven representative OSR methods are evaluated under two backbone strategies: frozen and full fine-tuning (full FT). Methods that require additional training are indicated in each group.

This study presents a systematic evaluation of open-set recognition methods on WiFi BVP features. As illustrated in Figure 2, our framework consists of two stages. In the first stage, a CNN–GRU backbone is pre-trained on the five known gesture classes using a standard Softmax classifier and cross-entropy loss. This stage produces a backbone that extracts discriminative 128-dimensional embeddings from BVP samples. No unknown-class data is involved.

In the second stage, we evaluate seven representative OSR methods on top of this pre-trained backbone: MSP, OpenMax, Energy, ODIN, K-NN, prototype, and rejection head. These methods cover the main mechanism categories in the OSR literature, including logit-based scoring, distance-based rejection, and pseudo-unknown-based training. Some methods require additional training on top of the backbone (prototype and rejection head), while others are applied directly to the logits or embeddings without training. To examine the effect of backbone adaptation, we consider two strategies: (1) frozen, where the backbone parameters remain unchanged, and (2) full FT, where the backbone is fine-tuned together with the rejection mechanism (if any).

This study provides a controlled comparison of seven existing OSR methods under a unified backbone and evaluation protocol, with the goal of isolating the effect of the rejection mechanism from that of the feature extractor.

### 3.2. CNN–GRU Gesture Embedding Encoder

This study employs the CNN–GRU architecture originally proposed in Widar3.0 [10] as its feature extraction backbone. This architecture processes the spatial and temporal dimensions of BVP data sequentially: a 2D-CNN extracts per-frame spatial features, and a GRU models temporal dependencies across frames.

Input representation. Each BVP sample is a three-dimensional tensor $X\in R^{H\times W\times T}$, where *H* = 20 and *W* = 20 correspond to the velocity components along the *x*-axis and *y*-axis (ranging from −2 m/s to +2 m/s, quantized into 20 bins), and *T* = 16 is the number of temporal frames after interpolation to a uniform length. Denoting the BVP map at frame t as $X_{t}\in R^{20\times 20}$, the input sample consists of the frame sequence $\left\{X_{1},X_{2},\ldots ,X_{T}\right\}$.

Spatial feature extraction. Three convolutional blocks are applied independently to each frame $X_{t}$. Each block $\ell \in \left\{1,2,3\right\}$ consists of a 2D convolution with kernel size 3 × 3, followed by Batch Normalization (BN), ReLU activation, and 2 × 2 max pooling. The numbers of output channels for the three blocks are 16, 32, and 64, respectively. Let $W_{conv}^{\left(\ell \right)}$ and $b_{conv}^{\left(\ell \right)}$ denote the convolution kernel and bias of block ℓ. The output feature map of block ℓ for frame *t* is computed as:

(1) $$G_{t}^{\left(\ell \right)}=MaxPool(ReLU(BN(W_{conv}^{\left(\ell \right)}*G_{t}^{\left(\ell -1\right)}+b_{conv}^{\left(\ell \right)})))$$

where $G_{t}^{\left(0\right)}=X_{t}$ and * denotes the 2D convolution operation. After the third convolutional block, an adaptive average pooling layer compresses the spatial dimensions to 1 × 1, and the resulting feature map is flattened to produce a frame-level feature vector $f_{t}\in R^{64}$. Processing all *T* = 16 frames yields the feature sequence $\left\{f_{1},f_{2},\ldots ,f_{T}\right\}$. These frame-level features are stacked along the temporal dimension to form the sequence tensor:

(2) $$F=Stack(f_{1},f_{2},\ldots ,f_{T})\in R^{B\times T\times 64}$$

Temporal modeling. The frame-level feature sequence is fed into a single-layer Gated Recurrent Unit (GRU) to capture the temporal dynamics of the gesture. The GRU maintains a hidden state $h_{t}\in R^{d_{h}}$ with $d_{h}$ = 128. At each time step *t*, the GRU updates its hidden state via the following gating mechanism:

(3) $$r_{t}=\sigma \;(W_{ir}f_{t}+W_{hr}h_{t-1}+b_{r})$$

(4) $$u_{t}=\sigma \;(W_{iu}f_{t}+W_{hu}h_{t-1}+b_{u})$$

(5) $${\tilde{h}}_{t}=tanh(W_{ih}f_{t}+W_{hh}(r_{t}⊙h_{t-1})+b_{h})$$

(6) $$h_{t}=(1-u_{t})⊙h_{t-1}+u_{t}⊙{\tilde{h}}_{t}$$

where $r_{t}$ and $u_{t}$ are the reset gate and update gate, ${\tilde{h}}_{t}$ is the candidate hidden state, σ denotes the Sigmoid activation, and ⊙ denotes element-wise multiplication. The weight matrices $W_{ir}$, $W_{iu}$, $W_{ih}\in R^{128\times 64}$ transform the input feature, and $W_{hr}$, $W_{hu}$, $W_{hh}\in R^{128\times 128}$ transform the previous hidden state. The bias vectors are $b_{r}$, $b_{u}$, $b_{h}\in R^{128}$. The final hidden state $h_{T}$ is taken as the embedding of the entire gesture sequence:

(7) $$z=h_{T}\in R^{128}$$

The resulting embedding ***z*** serves as the common feature representation for all evaluated OSR methods.

Closed-set pre-training. During the pre-training stage, a linear Softmax classifier is attached to the backbone. Given the embedding ***z***, the predicted probability for class *k* is:

(8) $${\hat{y}}_{k}=\frac{exp(w_{k}^{⊺}z+b_{k})}{\sum_{j=1}^{K}exp(w_{j}^{⊺}z+b_{j})}$$

where $w_{k}\in R^{128}$ and $b_{k}\in R$ are the classifier parameters for class *k*, and *K* = 5 is the number of known gesture classes. The backbone and classifier are jointly trained by minimizing the cross-entropy loss:

(9) $$L_{cls}=-\frac{1}{B}\sum_{i=1}^{B}log{\hat{y}}_{i,y_{i}}$$

where *B* is the batch size and $y_{i}$ is the ground-truth label. Algorithm 1 summarizes the complete pre-training procedure.
**Algorithm 1:** CNN–GRU Backbone Pre-Training for BVP Gesture Classification**Input:** Training set $D_{train}={\left\{(X_{i},y_{i})\right\}}_{i=1}^{N}$, number of known classes *K* = 5, embedding dimension *d* = 128, temporal frames *T* = 16, epochs *E* = 60, batch size *B*, learning rate $\eta =1\times 10^{-4}$.
**Output:** Pre-trained backbone parameters $\theta^{*}$. 1: Initialize backbone parameters θ randomly 2: **for** epoch = 1 to *E* **do** 3:    **for** each mini-batch ${\left\{(X_{b},y_{b})\right\}}_{b=1}^{B}$ from $D_{train}$ **do** 4:        **for** *t* = 1 to *T* **do** 5:            ${\;f}_{t}\;⟵\;FrameEncoder(X_{b}\left[:,:,t\right])$      ⊳2D−CNN:(B,1,20,20)⟶(B,64)
6:        **end for**
7:                  $F\;⟵\;Stack(f_{1},\ldots ,f_{T})$                                    ⊳(B,T,64) 8:                 $\;h_{T}\;⟵\;GRU(F)$                                               $⊳h_{T}:(1,B,d)$ 9:                 $z\;⟵\;Squeeze(h_{T})$                                            ⊳(B,d) 10:                $\hat{y}\;⟵Softmax(W_{cls}z+b_{cls})$                           ⊳(B,K) 11:                $L_{cls}⟵\;-\frac{1}{B}\sum_{b=1}^{B}log{\hat{y}}_{b,y_{b}}$ 12:                $\theta ⟵\;\theta -\;\eta \;\cdot \;\nabla_{\theta }L_{cls}$                                        ⊳Adam optimizer 13:    **end for** 14: **end for** 15: Save $\theta^{*}⟵\;\theta$


### 3.3. Evaluated OSR Methods

We evaluate seven representative OSR methods that cover the main mechanism categories in the literature: logit-based scoring (MSP, OpenMax, Energy, ODIN), distance-based rejection (K-NN, prototype), and pseudo-unknown-based training (rejection head). All methods operate on the same pre-trained CNN–GRU backbone, under either a frozen or fine-tuned configuration, so that the comparison isolates the effect of the rejection mechanism.

MSP (Maximum Softmax Probability). The simplest OSR baseline [17]. Given a test sample, the maximum Softmax probability is used as the acceptance score; samples with scores below a threshold are rejected as unknown. MSP requires no additional training.

OpenMax. OpenMax [18] extends the Softmax classifier by fitting a Weibull distribution to the penultimate-layer features of each known class. During inference, the activation vector is recalibrated by the Weibull tail probabilities, and the remaining probability mass is reallocated to an additional unknown class. We use a tail size of 20 for Weibull fitting, following the original OpenMax paper [18].

Energy-Based OOD Detection. The Energy method [19] uses the log-sum-exp of the logits as the anomaly score: $E(x)=-Tlog\sum_{j}exp(z_{j}/T)$, where $z_{j}$ are the logits and T is a temperature parameter. Higher energy indicates greater likelihood of being unknown. We use the standard setting T = 1 as in the original Energy paper [19].

ODIN. ODIN [20] applies temperature scaling to the logits and adds a small input perturbation to amplify the separation between known and unknown scores. We use a temperature of 1000 following the original ODIN paper [20], but disable input perturbation as its magnitude is difficult to calibrate on BVP features. The resulting score is the negative maximum Softmax probability after temperature scaling.

K-NN (Widar3.0). This method follows the K-NN-based outlier detection proposed in Widar3.0 [10]. For each test sample, the local density ratio $\rho ={LD}_{K}/{ELD}_{K}$ is computed, where ${LD}_{K}$ is the mean distance to its K nearest neighbors within the potential class, and ${ELD}_{K}$ is the average of the mean distances from those neighbors to their own K nearest neighbors. A higher ρ indicates a greater likelihood of being unknown. We use K = 20 following the parameter selection in Widar3.0 [10].

Prototype. Each known class is represented by a prototype vector in the embedding space. The rejection mechanism is based on the geometric property of L2-normalized Euclidean distance: samples far from all prototypes are rejected as unknown. To preserve the pre-trained embedding space that supports this geometric property, the backbone is kept frozen while only the prototype vectors are learned. At inference, the minimum distance from a test sample to all prototypes is used as the anomaly score; samples with distances above a threshold are rejected as unknown.

Rejection Head. A lightweight MLP is trained on top of the frozen backbone to output an anomaly score. Following the pseudo-unknown paradigm, we generate pseudo-unknown samples via Mixup between different known classes [29] and train the rejection head to assign high anomaly scores to these mixed samples while assigning low scores to known-class samples. These mixed samples are not treated as true unknowns; rather, they serve as auxiliary negative samples that encourage the rejection head to detect samples lying between known classes in the embedding space. Since the rejection head is an independent module trained on top of the frozen backbone and does not alter the closed-set classifier, the known-class decision boundaries remain unchanged. This approach is representative of OSR methods that train an auxiliary rejection module using generated or external outliers [22,23]. We use Mixup with α = 0.2, following common practice in pseudo-unknown generation.

## 4. Experiments

### 4.1. Experimental Setup

#### 4.1.1. Dataset

We conduct all experiments on the public Widar3.0 BVP dataset [10], which is a widely adopted benchmark for domain-independent WiFi gesture recognition. The dataset contains BVP features extracted from CSI measurements collected across multiple rooms, user positions, and body orientations. Each BVP sample is represented as a three-dimensional tensor $X\in R^{20\times 20\times T}$ encoding the power distribution over body-coordinate velocity components along the *x*-axis and *y*-axis. The temporal dimension *T* is normalized to 16 frames through linear interpolation. We select six representative gestures: Push and Pull, Sweep, Clap, Slide, Draw-O (Horizontal), and Draw-Zigzag (Horizontal). For simplicity, we refer to the latter two as Draw-O and Draw-Zigzag in the remainder of this paper. To avoid the bias of evaluating only a single unknown class, we adopt a leave-one-gesture-out protocol: each of the six gestures is alternately designated as the unknown class, while the remaining five serve as known classes. The dataset includes data from 15 users across three rooms with multiple body locations and orientations, yielding approximately 1873 valid BVP samples per gesture class after filtering corrupted files.

#### 4.1.2. Evaluation Protocol

(1) Domain-Disjoint Split: To rigorously evaluate generalization capability, we adopt a strict domain-disjoint split protocol. A domain is defined as a unique combination of (user ID, body location, body orientation). The unique domains are randomly split into training domains (70%) and testing domains (30%) using a fixed random seed. Since the split is performed at the domain level rather than the user level, the same user may appear in both training and testing, but only under different combinations of body location and orientation. For example, a sample from user 1 at location 1 with orientation 0 may be assigned to the training set, while another sample from user 1 at location 2 with orientation 1 may be assigned to the testing set. This ensures that no exact (user, location, orientation) tuple is shared between training and testing, but does not enforce user-level disjointness. The same split is used across all unknown-class configurations and all random seeds to ensure that all methods are evaluated on the same test set.

(2) Open-Set Protocol: We adopt a leave-one-gesture-out open-set protocol. In each configuration, one of the six gestures is designated as the unknown class, while the remaining five serve as known classes. During training, only samples from the five known classes are used. During testing, the model must either classify a sample into one of the five known classes, or reject it as unknown. For the three core unknown gestures (Push and Pull, Draw-O, Draw-Zigzag), we repeat all experiments with three random seeds (42, 123, 2024) to estimate variance. For the remaining three gestures (Sweep, Clap, Slide), we report a single run with seed 42 for trend analysis.

(3) Evaluation Metrics: We employ both threshold-independent and threshold-dependent metrics. For threshold-independent evaluation, we use AUROC (Area Under the Receiver Operating Characteristic curve), which measures the overall discriminability between known and unknown classes, and AUPRC (Area Under the Precision–Recall curve), which is more sensitive to class imbalance. We also report FPR80, the false-positive rate at 80% true-positive rate (i.e., the fraction of known-class samples wrongly rejected when 80% of unknown-class samples are correctly rejected). For threshold-dependent evaluation, we use known-class accuracy, the classification accuracy on known-class test samples; unknown detection rate, the proportion of unknown-class samples correctly rejected; and F1, the harmonic mean of precision and recall for the unknown class. The rejection threshold is selected on the training set to accept 95% of known-class samples. The training set contains only known-class samples, as no unknown-class data is used during training or threshold selection. This threshold is then fixed and applied to the test set for all threshold-dependent metrics.

### 4.2. Closed-Set Classifiers Fail Under Open-Set Conditions

We first establish a closed-set baseline by training the CNN–GRU backbone with a standard Softmax classifier on the five known gesture classes. Table 1 summarizes the classification performance across all six leave-one-gesture-out configurations. The model achieves training accuracies of 93.9–95.4% and testing accuracies of 76.9–84.3% across the six configurations, confirming that the CNN–GRU backbone effectively extracts discriminative features from BVP data.

However, when an unknown gesture is introduced during testing, the model consistently exhibits high-confidence misclassification. Across the six unknown gestures, 49.7–74.3% of unknown-class samples are misclassified into one of the five known classes with a Softmax confidence exceeding 0.8. Moreover, the mean Softmax confidence of unknown-class samples (0.760–0.834) is notably close to that of known-class samples (0.903–0.913), indicating that the model is not merely “guessing” but is genuinely overconfident in its incorrect predictions.

**Figure 3:**
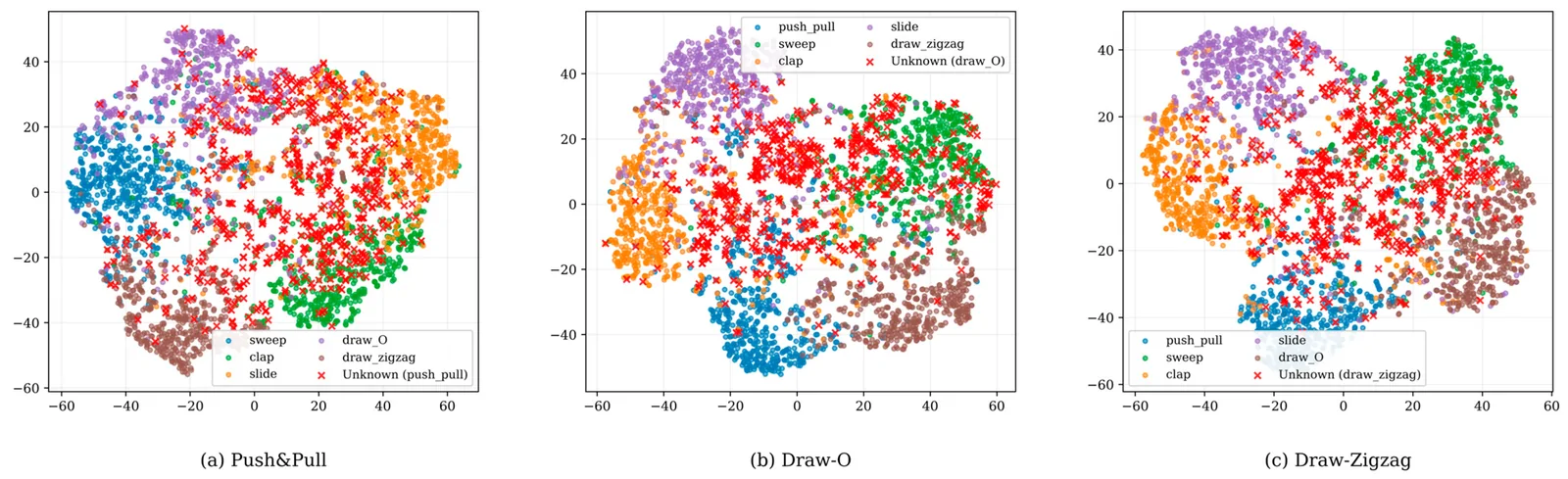
t-SNE visualization of the 128-dimensional embeddings learned by the closed-set Softmax backbone for three unknown-gesture configurations. Known classes are shown as colored dots, and the unknown class as red crosses. In all three configurations, known classes form distinct clusters, while unknown-class samples are largely interspersed within and around them. (**a**) Push and Pull, (**b**) Draw-O, (**c**) Draw-Zigzag.

To understand the source of this misclassification, we visualize the feature representations learned by the closed-set Softmax backbone. Figure 3 presents the t-SNE (t-distributed Stochastic Neighbor Embedding) projection of the 128-dimensional embeddings for three representative unknown-gesture configurations: Push and Pull, Draw-O, and Draw-Zigzag. In all three configurations, the five known classes form distinct, well-separated clusters, confirming that the backbone extracts discriminative features. However, the unknown-class samples (red crosses) are largely interspersed within and around the known-class clusters. The degree of overlap varies across configurations: for Draw-Zigzag (Figure 3c), the unknown samples overlap primarily with Draw-O and Sweep; for Draw-O (Figure 3b), they overlap with several known classes; and for Push and Pull (Figure 3a), they are spread across multiple known-class regions. This spatial overlap explains why the Softmax classifier assigns high confidence to incorrect predictions. In the learned feature space, unknown gestures are embedded close to certain known-class centers, and the Softmax normalization forces the model to distribute its probability mass among the known classes regardless of whether the input truly belongs to any of them.

**Figure 4:**
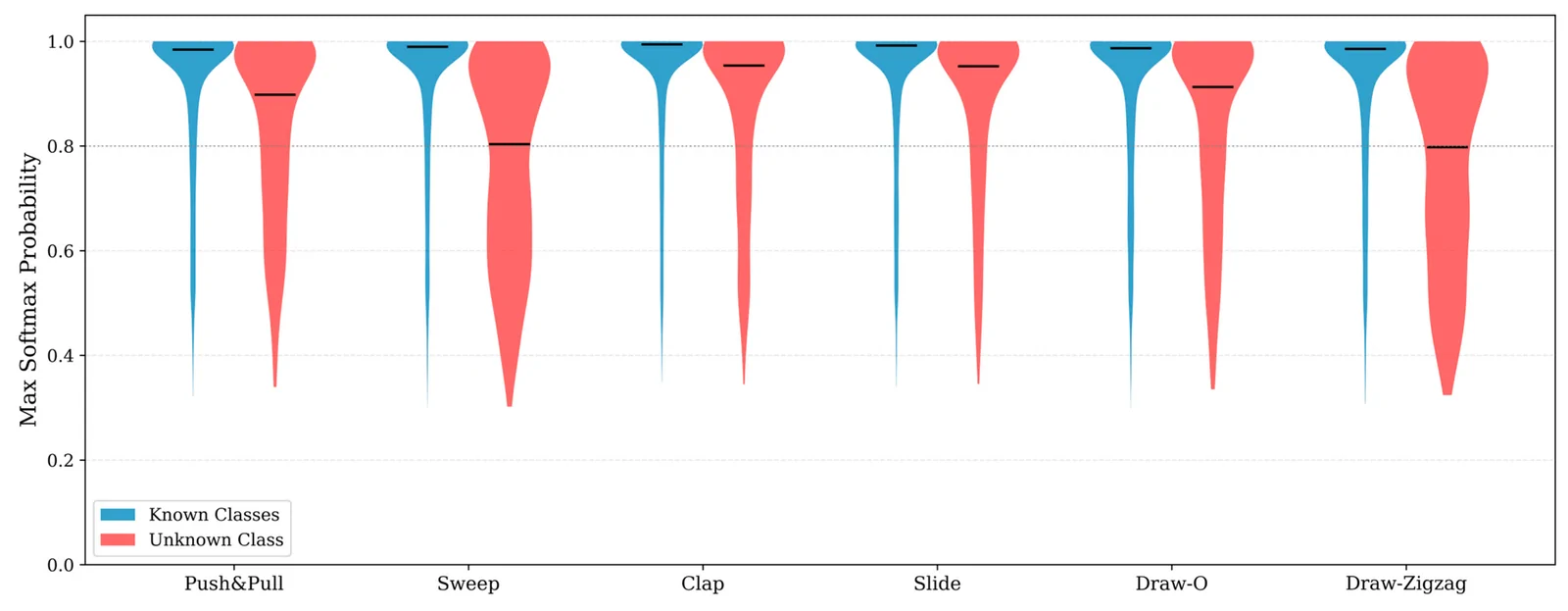
Violin plots of maximum Softmax probabilities for known-class (blue) and unknown-class (red) test samples across six unknown-gesture configurations. Black lines mark the median; the dashed lines represent the probability scale, with the 0.8 level indicating the confidence threshold.

Figure 4 further quantifies this behavior across all six unknown-gesture configurations using violin plots, which depict the full probability density of the maximum Softmax probabilities. For known classes (blue), the distributions are highly concentrated near 1.0, with narrow interquartile ranges, reflecting the classifier’s high confidence on in-distribution samples. For unknown classes (red), the distributions are substantially broader, with medians ranging from approximately 0.80 to 0.95 depending on the gesture. Importantly, despite their lower medians, each unknown-class distribution exhibits a heavy upper tail: a considerable fraction of unknown samples receive confidence scores exceeding 0.8, overlapping substantially with the known-class distributions. This overlap is most pronounced for Clap and Draw-Zigzag, where the unknown-class median approaches or falls below 0.8, whereas Push and Pull and Draw-O show relatively less overlap. These patterns are consistent with the high-confidence misclassification rates reported in Table 1, and they confirm that the closed-set Softmax classifier is systematically overconfident on unknown gestures across all evaluated configurations.

These findings demonstrate that closed-set classifiers, even when well trained on BVP features, consistently exhibit high-confidence misclassification on unknown gestures. The overconfidence is not a consequence of insufficient feature learning, since the backbone extracts highly discriminative features, as evidenced by the clear class separation across the three configurations in Figure 3. Rather, it reflects a structural limitation of the Softmax mechanism, whose forced probability normalization compels the model to distribute confidence among known classes even for inputs that belong to none of them. This motivates the evaluation of representative open-set recognition methods that can explicitly reject unknown gestures.

### 4.3. Rejection Mechanisms and Training Strategies: A Controlled Comparison

We evaluate seven representative OSR methods under two backbone strategies: frozen and full fine-tuning (full FT). The seven methods are maximum Softmax probability (MSP) [17], OpenMax [18], Energy-based OOD detection [19], ODIN [20], K-NN (Widar3.0) [10], prototype (Euclidean distance to class prototypes) [21], and rejection head [22,23]. The evaluation covers three core unknown gestures: Push and Pull, Draw-O, and Draw-Zigzag.

Table 2 and Table 3 summarize the threshold-independent performance under the frozen and full FT backbones, respectively. Under the frozen backbone, Energy and ODIN achieve the highest AUROC on Draw-Zigzag (0.835 and 0.836), while prototype performs best on Draw-O (0.740) and rejection head performs best on Push and Pull (0.736). Under full FT, Energy and ODIN again perform best on Draw-Zigzag (0.829 and 0.833), prototype is best on Draw-O (0.761), and Energy is best on Push and Pull (0.700). The AUPRC and FPR80 values show the same ranking as AUROC, confirming that these threshold-independent metrics are consistent with each other.

**Figure 5:**
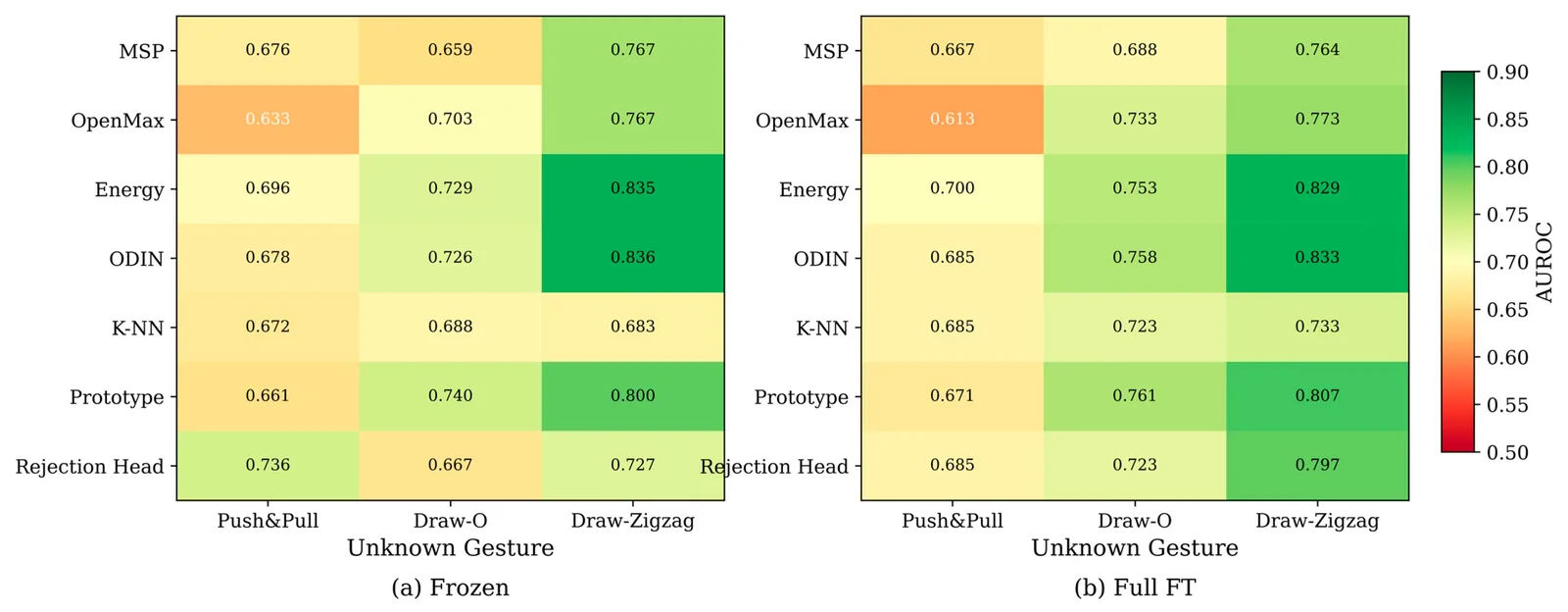
AUROC heatmap of seven OSR methods across three core unknown gestures under frozen and full FT backbones. Each cell shows the AUROC value (seed = 42). (**a**) Frozen, (**b**) full FT.

Figure 5 provides a heatmap overview of the AUROC values for all seven methods across the three core unknown gestures under both frozen and full FT backbones. The heatmap visually confirms that no single method achieves the highest AUROC across all three gestures. Under the frozen backbone, rejection head achieves the highest AUROC on Push and Pull (0.736), prototype on Draw-O (0.740), and Energy and ODIN on Draw-Zigzag (0.835 and 0.836). Under full FT, a similar pattern holds: Energy and ODIN are best on Draw-Zigzag (0.829 and 0.833), prototype on Draw-O (0.761), and Energy on Push and Pull (0.700). This inconsistency indicates that the optimal rejection mechanism depends on the unknown gesture rather than being universally superior.

To assess whether the differences between frozen and full FT are stable, we select four representative methods—Energy, ODIN, prototype, and rejection head—that cover the main mechanism categories in the OSR literature (logit-based scoring, distance-based rejection, and pseudo-unknown-based training). We repeat the evaluation for these four methods using three random seeds (42, 123, 2024). Table 4 reports the mean and standard deviation of AUROC. The difference between frozen and full FT is small for most methods: for Energy, the maximum difference across gestures is 0.017; for ODIN, it is 0.020; for prototype, it is 0.010. The only exception is rejection head, which shows a larger difference on Draw-O (0.624 frozen vs. 0.688 full FT), but this method also exhibits the largest variance across seeds (std up to 0.049), making the difference less reliable.

**Figure 6:**
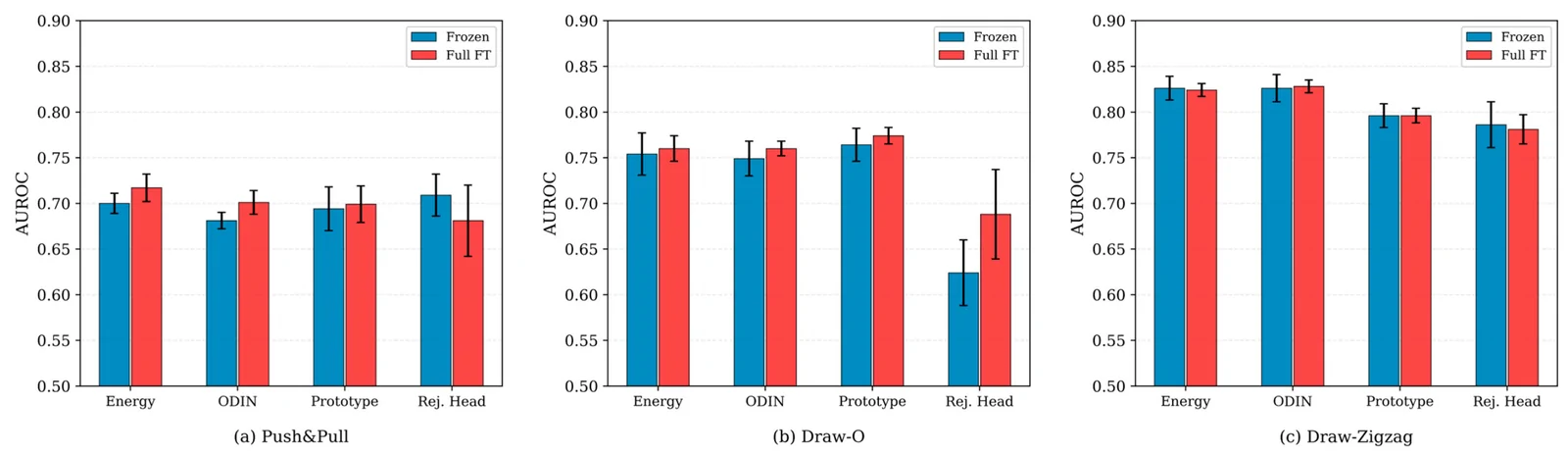
AUROC of four representative OSR methods under frozen and full FT backbones across three unknown gestures. The bars represent the mean AUROC, and error bars indicate the standard deviation over three random seeds. (**a**) Push and Pull, (**b**) Draw-O, (**c**) Draw-Zigzag.

Figure 6 visualizes these results as grouped bar charts for the three unknown gestures. Three observations are evident. First, the frozen and full FT bars are nearly identical for most methods, confirming that fine-tuning does not systematically improve open-set performance. Second, the error bars for Energy, ODIN, and prototype are small (std ≤ 0.023), while rejection head exhibits noticeably larger variance, particularly on Draw-O (std = 0.049). Third, the relative ranking of methods changes across unknown gestures: rejection head is best on Push and Pull under frozen, prototype is best on Draw-O under both strategies, and Energy and ODIN are best on Draw-Zigzag. This inconsistency reinforces that no single method dominates universally.

These results challenge the common assumption that fine-tuning a pre-trained backbone always improves open-set performance. On the CNN–GRU backbone, the frozen and fine-tuned configurations yield comparable results, with differences typically within the run-to-run variation of approximately ±0.02 AUROC. This suggests that for BVP features, the choice of rejection method and the specific unknown gesture matter more than whether the backbone is fine-tuned.

### 4.4. Closed-Set Misclassification Is Associated with Open-Set Difficulty

The results in Section 4.3 show that open-set performance varies substantially across unknown gestures. For example, under the frozen backbone, the average AUROC across seven OSR methods ranges from 0.65 for Clap to 0.77 for Draw-Zigzag. This raises a question: what factors are associated with the difficulty of rejecting an unknown gesture? In this section, we examine two candidate metrics: the geometric separation between the unknown class and the known classes in the embedding space, and the degree to which the closed-set classifier is confused by the unknown class.

We compute two metrics for each of the six unknown gestures under the frozen CNN–GRU backbone. The first is the centroid gap, defined as the Euclidean distance between the centroid of the unknown-class embeddings and the nearest known-class centroid. This metric is computed directly from the embedding space and does not depend on any OSR method or the Softmax classifier. It reflects the geometric separation between the unknown class and the known classes. The second is the closed-set high-confidence misclassification rate, defined as the proportion of unknown-class samples that are classified into a known class with a Softmax confidence exceeding 0.8. This metric captures how confidently the closed-set classifier misclassifies the unknown gesture.

**Figure 7:**
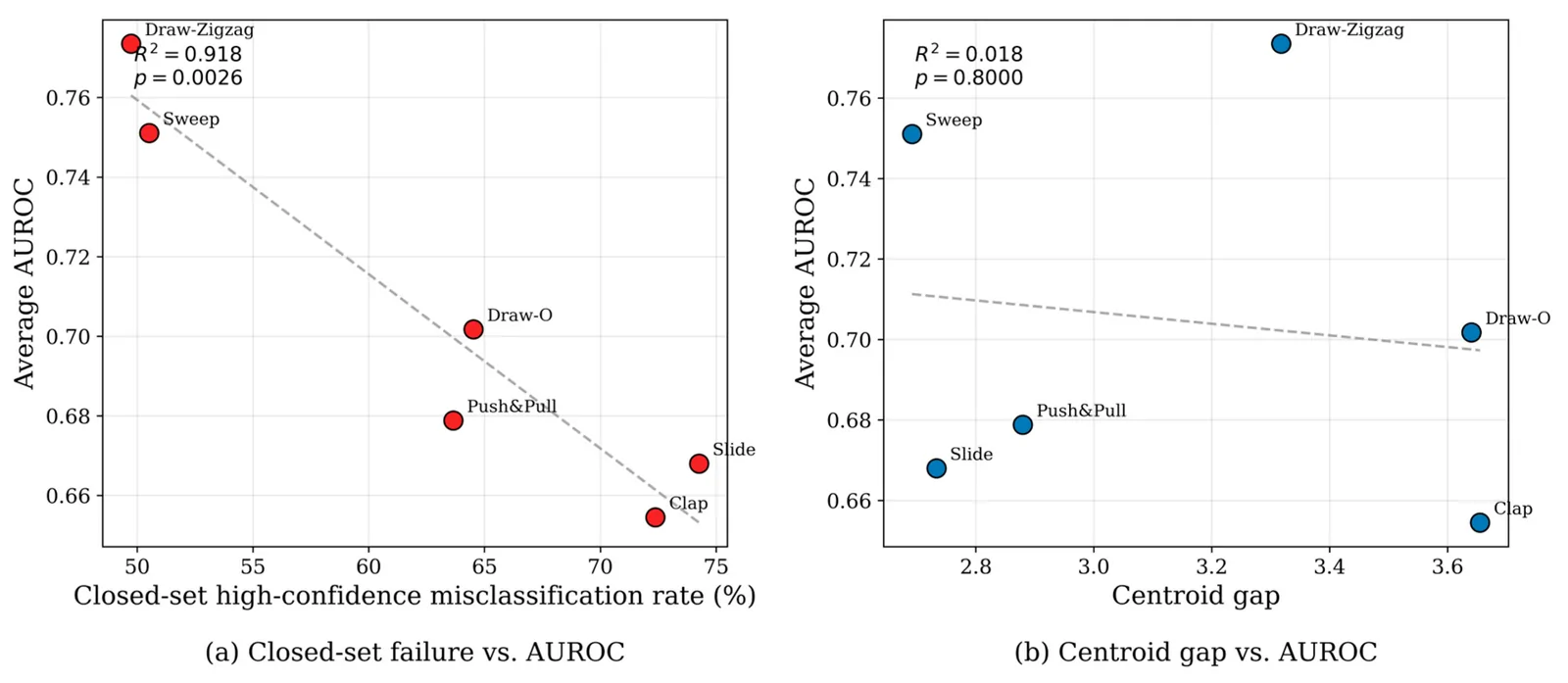
Relationship between open-set AUROC and two candidate difficulty metrics across six unknown gestures (frozen CNN–GRU backbone). The dashed lines represent the linear regression fits. (**a**) Closed-set high-confidence misclassification rate vs. AUROC ($R^{2}$ = 0.918, *p* = 0.0026). (**b**) Centroid gap vs. AUROC ($R^{2}$ = 0.018, *p* = 0.80).

The association is assessed using linear regression. For each metric, we fit a linear model predicting AUROC from the metric and report the coefficient of determination ($R^{2}$) and the *p*-value for the slope. A *p*-value below 0.05 is considered statistically significant. Figure 7 plots the average AUROC (over seven OSR methods) against each of these two metrics. The closed-set high-confidence misclassification rate shows a strong negative association with AUROC ($R^{2}$ = 0.918, *p* = 0.0026), whereas the centroid gap shows no significant association ($R^{2}$ = 0.018, *p* = 0.80). Table 5 summarizes the correlation statistics.

These observations suggest that open-set difficulty may be more closely related to the behavior of the classifier near the decision boundaries than to the geometric structure of the embedding space. When the closed-set classifier confidently misclassifies unknown samples into known classes, those samples are likely to lie close to the decision boundaries of the known classes. Consequently, rejection mechanisms—whether based on logits, distances, or additional training—may struggle to separate them. In contrast, a large centroid gap does not guarantee that unknown samples are far from decision boundaries; the samples could be widely dispersed and overlap with known-class regions near the boundaries.

We note that this analysis is based on six unknown gestures, three of which were evaluated with a single random seed. While the observed association is statistically significant, the small sample size limits the generalizability of this finding. Extending the analysis to more unknown gestures and multiple seeds is left for future work.

### 4.5. Multi-Objective Evaluation and Deployment Guidelines

While the threshold-independent metrics in Section 4.3 measure overall discriminability, they do not capture the trade-off between accepting known-class samples and rejecting unknown ones at a specific operating point. In practical deployments, the choice of rejection threshold depends on the relative cost of false acceptances (known samples wrongly rejected) and false rejections (unknown samples wrongly accepted). To provide actionable guidance, we analyze the performance of the evaluated OSR methods across multiple operating points.

Threshold selection. Throughout this study, the rejection threshold is selected on the training set to accept 95% of known-class samples. Importantly, this threshold is determined without using any unknown-class data. The same threshold is then fixed and applied to the test set for all threshold-dependent metrics. To examine the sensitivity of our results to this choice, we also report performance at 90%, 80%, and 70% acceptance rates. Our primary metric is AUROC, which is threshold-independent; the threshold-dependent metrics are reported only to analyze the operating-point trade-off. The consistent trends across acceptance rates indicate that our conclusions are not an artifact of a particular threshold.

Operating point analysis. Table 6 shows the trade-off for the frozen backbone on Draw-Zigzag, which is representative of the observed trends. At the conservative 95% acceptance rate, Energy achieves a known-class accuracy of 92.0% and an unknown detection rate of 40.5%. Lowering the acceptance rate to 80% increases the unknown detection rate to 76.0% while reducing known-class accuracy to 75.0%; the F1 score peaks at 80% acceptance (0.505). This pattern holds across other methods and gestures: increasing the willingness to reject known samples substantially improves unknown detection at the cost of known-class accuracy. The optimal operating point therefore depends on the deployment requirement.

**Figure 8:**
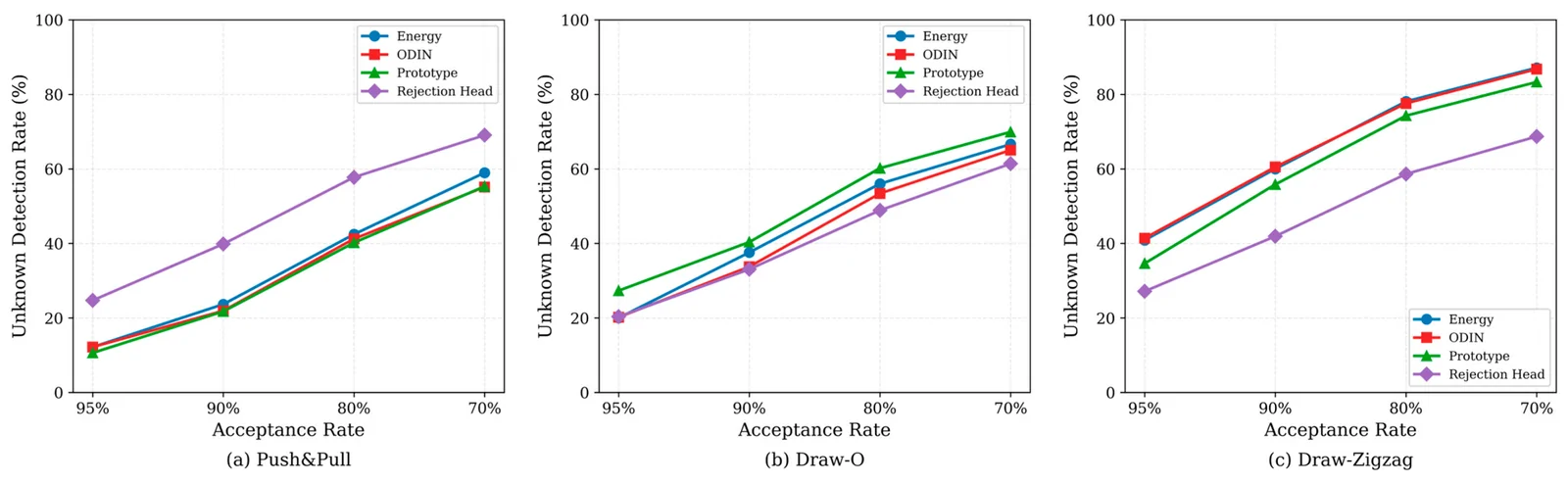
Unknown detection rate (UDR) as a function of the acceptance rate for four representative OSR methods under the frozen backbone. Each curve corresponds to one method; lower acceptance rates indicate more aggressive rejection of known-class samples. (**a**) Push and Pull, (**b**) Draw-O, (**c**) Draw-Zigzag.

Figure 8 visualizes the operating-point trade-off between acceptance rate and UDR for four representative methods under the frozen backbone. Across all three unknown gestures, decreasing the acceptance rate from 95% to 70% consistently increases the UDR for every method, at the cost of reduced known-class accuracy (as quantified in Table 6). The relative ranking of methods varies across gestures: on Push and Pull, rejection head achieves the highest UDR across all acceptance rates; on Draw-O, prototype and Energy are competitive while rejection head is noticeably lower; and on Draw-Zigzag, Energy and ODIN are the top performers. These trends reinforce two observations: (1) no single method dominates across all unknown gestures, and (2) the choice of operating point has a larger effect on UDR than the choice of method itself. This highlights the importance of considering deployment constraints when selecting both a rejection method and an operating threshold.

**Figure 9:**
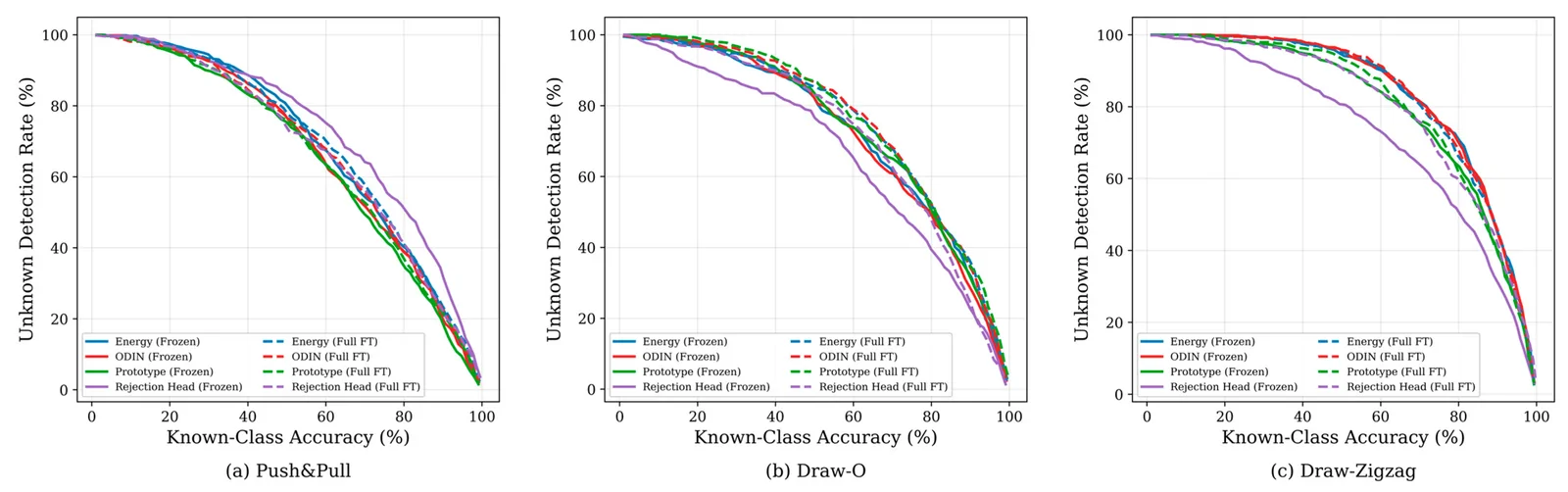
Pareto frontier of known-class accuracy versus unknown detection rate for four representative OSR methods under frozen and full FT backbones, across three unknown gestures. Each curve corresponds to one method–backbone combination, generated by sweeping the rejection threshold. (**a**) Push and Pull, (**b**) Draw-O, (**c**) Draw-Zigzag.

Pareto frontier. Figure 9 presents the Pareto frontier of known-class accuracy versus unknown detection rate for four representative methods (Energy, ODIN, prototype, and rejection head) under both frozen and full FT backbones, across the three core unknown gestures. Each curve is generated by sweeping the rejection threshold over its full range; each point on a curve corresponds to one threshold value. This representation complements AUROC, which summarizes the same information as a single scalar. The frontier shows that no single method dominates across the entire trade-off curve. Energy and ODIN generally achieve higher unknown detection rates for a given known-class accuracy, while prototype and rejection head offer competitive performance in specific regions. The frontier also reveals that the differences between methods are modest compared to the variation induced by the operating point itself.

Deployment observations (limited to our setting). Based on the operating point analysis above, we summarize a few observations that may inform method selection under our experimental setting (CNN–GRU backbone, Widar3.0 BVP, six evaluated unknown gestures, 95% acceptance rate unless otherwise stated) as shown in Table 7. These observations are trends within this study and should not be interpreted as general deployment rules.

- When known-class accuracy is prioritized (e.g., ≥90%), Energy under full FT tends to provide a favorable balance across the six unknown gestures evaluated;
- When unknown detection is prioritized (e.g., safety-critical scenarios), Energy or rejection head under the frozen backbone tend to achieve higher unknown detection rates on the gestures we evaluated;
- When F1 is used as a balanced metric, Energy under either backbone is consistently among the top performers in our setting.

These observations are limited to the CNN–GRU backbone, the Widar3.0 BVP dataset, and the six unknown gestures evaluated in this study. Different backbones, datasets, or unknown gestures may favor different methods. We therefore do not claim a universal recommendation; instead, we provide these trends as a starting point for further investigation.

### 4.6. Discussion

Our experiments on the Widar3.0 BVP dataset lead to four main observations. First, closed-set Softmax classifiers consistently misclassify unknown gestures with high confidence (49.7–74.3% across six unknown gestures). This is not a consequence of poor feature learning: the same backbone achieves 76.9–84.3% accuracy on known classes, and the known-class embeddings form distinct clusters. Rather, it reflects the structural limitation of the Softmax normalization, which forces probability mass onto known classes even for inputs that belong to none of them.

Second, across seven OSR methods and two backbone strategies, we find that no single method consistently outperforms all others across unknown gestures. Energy and ODIN generally achieve the highest AUROC on Draw-Zigzag, prototype performs best on Draw-O, and rejection head is competitive on Push and Pull. Moreover, the difference between frozen and fine-tuned backbones is small (typically within ±0.02 AUROC, with the exception of rejection head on Draw-O). This challenges the common assumption that fine-tuning a pre-trained backbone always improves open-set performance.

Third, we observe a strong association between the closed-set high-confidence misclassification rate and open-set AUROC ($R^{2}$ = 0.918, *p* = 0.0026), whereas the geometric separation between unknown and known classes (centroid gap) shows no significant association ($R^{2}$ = 0.018, *p* = 0.80). This suggests that open-set difficulty may be more closely related to classifier-level confusion than to the geometric structure of the embedding space. In other words, a large centroid distance does not guarantee that unknown samples lie far from decision boundaries; they may be dispersed and overlap with known-class regions near the boundaries. This finding aligns with the broader observation that Softmax-based classifiers are overconfident on out-of-distribution inputs, and it indicates that improving open-set recognition on BVP features may require addressing classifier uncertainty rather than merely increasing geometric separation.

Fourth, our multi-objective analysis shows that the operating point has a larger effect on the trade-off between known-class accuracy and unknown detection rate than the choice of method. At the 95% acceptance rate, most methods achieve an unknown detection rate between 30% and 45% on Draw-Zigzag, but lowering the acceptance rate to 80% increases this to 70–76% at the cost of 15–20% of known-class accuracy. This trade-off is inherent to threshold-based rejection and should be considered when selecting a method for deployment.

Relationship to prior work. Widar3.0 [10] first introduced a K-NN-based outlier detection algorithm for BVP features and reported an AUROC of 0.81 on their outlier detection task. Our results are consistent with that finding: K-NN achieves an AUROC of 0.68–0.74 in our setting, which is lower than Widar3.0’s reported value but comparable given the differences in unknown-class difficulty and evaluation protocol. Importantly, our study extends Widar3.0’s analysis in three ways: (1) we evaluate seven OSR methods rather than one, (2) we consider six unknown gestures instead of a single outlier set, and (3) we analyze the factors associated with open-set difficulty. In the broader OSR literature, methods such as Energy, ODIN, and OpenMax have been shown to outperform MSP on image benchmarks [18,19,20]. Our results on BVP features are partially consistent: Energy and ODIN achieve the highest AUROC on Draw-Zigzag, but their advantage over MSP is smaller on other gestures. This suggests that the relative ranking of OSR methods may depend on the feature space and the unknown-class configuration.

Limitations. Several limitations should be noted. First, all experiments are conducted on the CNN–GRU backbone, which is the standard architecture introduced in Widar3.0 for BVP-based gesture recognition. This controlled setting allows us to isolate the effect of rejection mechanisms and training strategies from the effect of backbone architecture. Recent works have explored alternative backbones for BVP features, such as graph convolutional networks, Transformers, and hybrid architectures. Whether the observed association between closed-set misclassification and open-set difficulty holds on these backbones remains an open question and is left for future work. Second, the analysis of difficulty factors is based on six unknown gestures, three of which were evaluated with a single random seed. Although the observed association is statistically significant, the small sample size warrants caution. Third, we did not perform an extensive hyperparameter search for fine-tuning strategies; it is possible that other learning rates or regularization settings could improve the fine-tuned results. Fourth, our study focuses on horizontal-plane BVP features; gestures with significant vertical motion may behave differently. Finally, we did not include certain OSR methods such as contrastive learning from scratch, which trains its own feature extractor and therefore falls outside our controlled comparison framework.

Future work. Several directions emerge from this study. First, extending the evaluation to other backbones (e.g., Transformer, GCN) and other WiFi sensing datasets would help validate the generalizability of our observations. Second, developing OSR methods that explicitly target classifier-level confusion—rather than relying on geometric separation—may lead to more effective rejection on BVP features. Third, the Mixup-generated pseudo-unknowns used in the rejection head are convex combinations of known classes and may not fully approximate the distribution of real unknown gestures. Exploring pseudo-unknown generation strategies that better approximate real unknown gestures could improve the stability of rejection heads. Finally, investigating the relationship between closed-set misclassification and open-set difficulty across a larger number of unknown classes would strengthen the statistical evidence for this association.

## 5. Conclusions

In this paper, we presented an empirical study of open-set gesture recognition on WiFi BVP features. Using the CNN–GRU backbone from Widar3.0, we systematically evaluated seven representative OSR methods under two backbone strategies (frozen and full fine-tuning) across six unknown gestures and three random seeds.

Our experiments yielded four main findings. First, closed-set Softmax classifiers consistently misclassify unknown gestures with high confidence (49.7–74.3% across six gestures), even though the same backbone achieves 76.9–84.3% accuracy on known classes. This indicates that the open-set vulnerability is not a consequence of insufficient feature learning, but a structural limitation of the Softmax normalization.

Second, across seven OSR methods and two backbone strategies, no single method consistently outperforms all others across unknown gestures. Energy and ODIN generally achieve the highest AUROC on Draw-Zigzag, prototype performs best on Draw-O, and rejection head is competitive on Push and Pull. The difference between frozen and fine-tuned backbones is small (typically within ±0.02 AUROC), challenging the common assumption that fine-tuning always improves open-set performance.

Third, we observed a strong association between the closed-set high-confidence misclassification rate and open-set AUROC ($R^{2}$ = 0.918, *p* = 0.0026), whereas the geometric separation between unknown and known classes (centroid gap) showed no significant association ($R^{2}$ = 0.018, *p* = 0.80). This suggests that open-set difficulty may be more closely related to classifier-level confusion than to the geometric structure of the embedding space.

Fourth, our multi-objective analysis showed that the operating point has a larger effect on the trade-off between known-class accuracy and unknown detection rate than the choice of method. At the 95% acceptance rate, most methods achieve an unknown detection rate between 30% and 45% on Draw-Zigzag, but lowering the acceptance rate to 80% increases this to 70–76% at the cost of 15–20% of known-class accuracy.

These findings suggest that improving open-set recognition on BVP features may require addressing classifier-level uncertainty rather than merely increasing geometric separation. Future work should extend the evaluation to other backbones and datasets, explore OSR methods that explicitly target classifier confusion, and develop pseudo-unknown generation strategies that better approximate real unknown gestures.

## Figures and Tables

**Table 1:** Closed-set classification performance of CNN–GRU + Softmax.

Unknown Gesture	Training Acc	Testing Acc	Unknown High-Confidence (>0.8) Misclassification Rate	Unknown Mean Confidence	Known Mean Confidence
Push and Pull	94.41%	77.95%	63.65%	0.831	0.905
Sweep	95.07%	81.79%	50.52%	0.766	0.913
Clap	96.00%	82.46%	72.38%	0.865	0.923
Slide	95.47%	84.29%	74.26%	0.872	0.918
Draw-O	95.44%	76.94%	64.52%	0.834	0.903
Draw-Zigzag	95.32%	83.21%	49.74%	0.760	0.904

**Table 2:** Threshold-independent OSR performance under the frozen backbone (seed = 42). Best AUROC per unknown gesture is highlighted in bold.

Unknown Gesture	Method	AUROC	AUPRC	FPR80
Push & Pull	MSP	0.6760	0.2557	0.5374
	OpenMax	0.6328	0.2198	0.5970
	Energy	0.6957	0.2814	0.4956
	ODIN	0.6780	0.2646	0.5259
	K-NN (Widar3.0)	0.6717	0.3021	0.5751
	Prototype	0.6614	0.2429	0.5468
	**Rejection Head**	**0.7361**	0.3467	0.4619
Draw-O	MSP	0.6587	0.2511	0.5535
	OpenMax	0.7032	0.3157	0.5242
	Energy	0.7290	0.3319	0.4901
	ODIN	0.7262	0.3166	0.4744
	K-NN (Widar3.0)	0.6883	0.3177	0.5632
	**Prototype**	**0.7400**	0.3412	0.4741
	Rejection Head	0.6667	0.2670	0.5364
Draw-Zigzag	MSP	0.7671	0.3543	0.3852
	OpenMax	0.7669	0.3523	0.4093
	Energy	0.8352	0.4564	0.2814
	**ODIN**	**0.8356**	0.4553	0.2828
	K-NN (Widar3.0)	0.6830	0.2857	0.5437
	Prototype	0.7999	0.4071	0.3448
	Rejection Head	0.7269	0.3364	0.4887

**Table 3:** Threshold-independent OSR performance under the full FT backbone (seed = 42). Best AUROC per unknown gesture is highlighted in bold.

Unknown Gesture	Method	AUROC	AUPRC	FPR80
Push & Pull	MSP	0.6668	0.2573	0.5413
	OpenMax	0.6135	0.2280	0.6618
	**Energy**	**0.6996**	0.2959	0.5134
	ODIN	0.6852	0.2812	0.5329
	K-NN (Widar3.0)	0.6849	0.2943	0.5535
	Prototype	0.6712	0.2627	0.5569
	Rejection Head	0.6854	0.2910	0.5444
Draw-O	MSP	0.6878	0.2675	0.5019
	OpenMax	0.7329	0.3360	0.4584
	Energy	0.7532	0.3523	0.4089
	ODIN	0.7584	0.3511	0.4107
	K-NN (Widar3.0)	0.7227	0.3252	0.4709
	**Prototype**	**0.7611**	0.3802	0.4309
	Rejection Head	0.7231	0.2967	0.4472
Draw-Zigzag	MSP	0.7638	0.3162	0.3772
	OpenMax	0.7729	0.3524	0.3608
	Energy	0.8289	0.4360	0.2933
	**ODIN**	**0.8331**	0.4378	0.2860
	K-NN (Widar3.0)	0.7330	0.3474	0.4922
	Prototype	0.8071	0.4113	0.3372
	Rejection Head	0.7973	0.4404	0.3521

**Table 4:** Mean ± std AUROC over three random seeds for four representative methods.

Method	Backbone	Push & Pull	Draw-O	Draw-Zigzag
Energy	Frozen	0.700 ± 0.011	0.754 ± 0.023	0.826 ± 0.013
	Full FT	0.717 ± 0.015	0.760 ± 0.014	0.824 ± 0.007
ODIN	Frozen	0.681 ± 0.009	0.749 ± 0.019	0.826 ± 0.015
	Full FT	0.701 ± 0.013	0.760 ± 0.008	0.828 ± 0.007
Prototype	Frozen	0.694 ± 0.024	0.764 ± 0.018	0.796 ± 0.013
	Full FT	0.699 ± 0.020	0.774 ± 0.009	0.796 ± 0.008
Rejection Head	Frozen	0.709 ± 0.023	0.624 ± 0.036	0.786 ± 0.025
	Full FT	0.681 ± 0.039	0.688 ± 0.049	0.781 ± 0.016

**Table 5:** Association between candidate difficulty metrics and open-set AUROC across six unknown gestures (frozen CNN–GRU backbone).

Metric	$R^{2}$	*p*-Value
Closed-set high-confidence misclassification rate	0.918	0.0026
Centroid gap (embedding geometry)	0.018	0.80

**Table 6:** Operating point analysis for the frozen backbone on Draw-Zigzag (representative, seed = 42). Known-Acc = known-class accuracy, UDR = unknown detection rate. Similar trends were observed across the three random seeds.

Acceptance Rate	Metric	Energy	ODIN	Prototype	Rejection Head
95%	Known-Acc	92.0%	92.2%	92.9%	91.6%
	UDR	40.5%	38.8%	32.9%	37.7%
	F1	0.449	0.436	0.391	0.421
90%	Known-Acc	86.2%	86.9%	86.1%	86.3%
	UDR	57.9%	55.7%	49.9%	55.7%
	F1	0.511	0.497	0.455	0.497
80%	Known-Acc	75.0%	75.5%	73.5%	75.5%
	UDR	76.0%	73.4%	71.8%	73.4%
	F1	0.505	0.496	0.472	0.496
70%	Known-Acc	64.2%	65.6%	63.1%	65.6%
	UDR	88.0%	81.6%	83.5%	81.6%
	F1	0.480	0.462	0.454	0.462

**Table 7:** Observed method preferences across the six evaluated unknown gestures under different deployment constraints in our setting (CNN–GRU backbone, Widar3.0 BVP).

Deployment Constraint	Observed Preference	Backbone
Known-Acc prioritized (≥90%)	Energy	Full FT
Unknown detection prioritized	Energy/Rejection Head	Frozen
Balanced F1	Energy	Frozen or Full FT

## Data Availability

The original contributions presented in this study are included in the article. Further inquiries can be directed to the corresponding author upon reasonable request.

## Abbreviations

The following abbreviations are used in this manuscript:

AUROC	Area Under the Receiver Operating Characteristic Curve
AUPRC	Area Under the Precision–Recall Curve
BN	Batch Normalization
BVP	Body-Coordinate Velocity Profile
CNN	Convolutional Neural Network
CSI	Channel State Information
FTR80	False-Positive Rate at 80% True-Positive Rate
Full FT	Full Fine-Tuning
GRU	Gated Recurrent Unit
MSP	Maximum Softmax Probability
OSR	Open-Set Recognition
ReLU	Rectified Linear Unit
t-SNE	t-distributed Stochastic Neighbor Embedding
